# CRAB-RSS: development and validation of a single-center risk stratification model for carbapenem-resistant *Acinetobacter baumannii* in ICU patients based on mechanical ventilation, carbapenem exposure, and hospitalization duration

**DOI:** 10.3389/fcimb.2025.1659853

**Published:** 2025-11-06

**Authors:** Kun Li, Jie Wang, Long Li

**Affiliations:** Department of Clinical Laboratory Medicine, Suining Central Hospital, Suining, Sichuan, China

**Keywords:** carbapenem-resistant Acinetobacter baumannii, risk stratification model, intensive care unit, antimicrobial resistance, infection control

## Abstract

**Purpose:**

This study aimed to develop and validate the Carbapenem-Resistant *Acinetobacter baumannii* Risk Scoring System (CRAB-RSS), a novel predictive model designed to assess the risk of carbapenem-resistant *A. baumannii* (CRAB) infection in intensive care unit (ICU) patients.

**Methods:**

A retrospective cohort analysis was performed on 412 patients (315 with CRAB and 97 with carbapenem-susceptible *A.baumannii* [CSAB]) from 2020 to 2024. Three independent risk factors were identified: mechanical ventilation (adjusted odds ratio [aOR] = 3.2, 95% confidence interval [CI]: 1.8–5.6), prior carbapenem exposure (≥48 hours; aOR = 1.89, 95% CI: 1.32–2.71), and hospitalization duration exceeding 14 days (aOR = 1.67, 95% CI: 1.25–2.23).

**Results:**

The model demonstrated robust discriminative ability, with an area under the receiver operating characteristic curve (AUROC) of 0.887 in the derivation cohort and 0.918 in the validation cohort, along with satisfactory calibration (Brier score: 0.094 versus 0.088). Its performance was significantly superior to that of the Sequential Organ Failure Assessment (SOFA) score (ΔAUROC = +0.21).

**Conclusions:**

CRAB-RSS is a quantitative risk stratification tool derived from a single-center cohort for CRAB infections in intensive care unit patients, demonstrating superior performance to SOFA in local validation. External multicenter validation is warranted before broad clinical implementation. Its innovative features include: (1) a fixed-weighting design (e.g., assigning a baseline score of 2 points for mechanical ventilation), and (2) reliance on only three readily obtainable clinical variables to complete the assessment. Decision curve analysis revealed that the application of CRAB-RSS could reduce unnecessary carbapenem use by 28%–42% across probability thresholds of 10%–30%, with a maximum reduction of 38% achieved at the 20% threshold.

## Introduction

The *Acinetobacter baumannii complex*(ABC) has emerged as a critical hospital-associated pathogen that poses a significant threat to patients in intensive care units (ICUs) worldwide. Its notable environmental persistence, capacity for biofilm formation on medical devices and surfaces, and rapid acquisition of antimicrobial resistance genes facilitate its role in causing severe healthcare-associated infections (HAIs), including ventilator-associated pneumonia (VAP), bloodstream infections (BSIs), catheter-associated urinary tract infections (CAUTIs), surgical site infections (SSIs), and meningitis. Critically ill ICU patients, who are often immunocompromised and subjected to invasive procedures and prolonged hospital stays, are particularly vulnerable to these infections, which are associated with substantial morbidity and mortality (Joly-Guillou, 2005; Munoz-Price and Weinstein, 2008).

Surveillance data indicate considerable geographic variation in CRAB prevalence. In China, rates surged from 39.0% in 2005 to 79.0% in 2019 (chinet), while our study at Suining Central Hospital (2020-2024) documented a CRAB rate of 76.5% among ICU patients—strikingly higher than the hospital-wide rate of 29.8%. This discrepancy underscores the disproportionate burden within critical care settings and highlights the urgent need for targeted intervention strategies.

High rates are also prevalent across Asia, including Thailand (83%), India (87%), South Korea (88%), and China (75%-78%). In Europe, countries like Greece, Turkey, and Croatia report rates ≥50%, while Italy documented an increase ICU incidence density from 1.1 per 1, 000 patient-days (2006-2007) to 3.0 (2012-2013). Latin America faces severe challenges, with Brazil reporting rates as high as 90%. Conversely, Japan (<5%) and Australia (6.5%) exhibit lower rates (Boutzoukas and Doi, 2025). Data from the United States indicates that 29.9% of ICUs reported carbapenem-non-susceptible *A. baumannii* isolates based on a 2018–2020 surveillance study, highlighting its ongoing burden (Sader et al., 2022). Estimating the true impact in many low- and middle-income countries remains challenging due to inadequate surveillance, suboptimal infection control, antibiotic misuse, and deficient healthcare infrastructure.

The formidable challenge posed by CRAB stems from its complex and evolving resistance mechanisms, particularly to carbapenems—broad-spectrum β-lactam antibiotics often considered drugs of last resort for Gram-negative infections. Carbapenem resistance is primarily mediated by the production of carbapenem-hydrolyzing enzymes (carbapenemases), notably OXA-type enzymes such as OXA-23, OXA-24, and OXA-58 (Asif et al., 2018). Additional mechanisms include alterations in efflux pumps (e.g., AdeABC) and outer membrane proteins reducing antibiotic permeability, coupled with acquired resistance genes for extended-spectrum β-lactamases (ESBLs) and aminoglycoside-modifying enzymes. Furthermore, the intrinsic ability of *A. baumannii* to form robust biofilms on abiotic surfaces (e.g., ventilators, catheters) provides a protective niche against antimicrobial agents and host defenses, thereby facilitating its persistence and transmission within the healthcare environment (Gedefie et al., 2021).

The emergence of CRAB represents a critical public health concern due to its devastating clinical outcomes. CRAB infections are notoriously difficult to treat and are associated with high mortality rates, ranging from 27.8% to 35% across various global regions. The CDC recognized CRAB as an “urgent threat” in its 2019 report. ICU patients face compounded risks attributable to underlying comorbidities, immunodeficiencies, and the frequent use of invasive devices (e.g., mechanical ventilation, central venous catheters, urinary catheters), which can serve as portals of entry and reservoirs for biofilm formation. Prior invasive procedures, prolonged hospitalization (>14 days), and antecedent exposure to broad-spectrum antimicrobials, particularly carbapenems, have been consistently identified as significant risk factors for CRAB acquisition and poor clinical outcomes (Peleg et al., 2008; Antunes et al., 2014; Garnacho-Montero et al., 2016; Brotfain et al., 2017).

Current strategies for managing suspected CRAB infections in the ICU often rely on drug susceptibility testing (resulting in diagnostic delays), empirical broad-spectrum therapy​ (contributing to resistance), or generic severity scores like SOFA or APACHE-II, which lack specificity for predicting CRAB risk. There is a critical unmet need for a dedicated, timely, and clinically practical tool to stratify ICU patients’ risk for CRAB infection to guide targeted interventions such as early contact isolation and optimized antibiotic stewardship. To address this gap, we conducted a retrospective cohort study (2020-2024) at Suining Central Hospital. To identify independent risk factors for CRAB infection in ICU patients and develop a retrospective risk-stratified prediction model (CRAB-RSS) based on these variables.This model is innovative in its (1) fixed weighting design(e.g., baseline assessment based on clinical status changes like mechanical ventilation) and (2) reliance on only three readily available clinical variables, enabling rapid point-of-care assessment to support infection prevention and rational antimicrobial use in the ICU setting. To address this gap, we conducted this retrospective cohort study with the following objectives: (1) to identify independent risk factors for CRAB infection; (2) to develop and validate a concise risk scoring system; and (3) to assess the practical utility of this model in clinical decision-making.

## Materials and methods

### Study design and population

This retrospective study evaluated all patients with *A. baumannii* infections occurring in the ICU as identified through the hospital information system (HIS) from January 1, 2020 to December 31, 2024 at the Suining Central Hospital, China. This study was approved by the Ethics Committee (KYLLKS20240192) of Suining Central Hospital.The committee waived the requirement for informed consent due to the use of de-identified retrospective data. All data were handled in accordance with the Declaration of Helsinki.Demographic, clinical, and microbiological data were collected for all cases. To minimize bias from repeated isolates, only the initial pathogen detection was considered for each patient, of the pathogen in each instance, while excluding cases with incomplete medical records. Patients were categorized into case (CRAB-positive, n=315) and control (carbapenem-susceptible [CSAB], n=97) groups based on initial isolate susceptibility testing, ensuring clear contrast for predictive modeling.

*A. baumannii* infection was defined according to CDC/NHSN surveillance criteria (2015)​, incorporating both microbiological and clinical criteria. Site-specific quantitative thresholds​ included: bronchoalveolar lavage (BAL) ≥10^4^ CFU/mL or protected specimen brush (PSB) ≥10³ CFU/mL for respiratory infections; ≥1 positive blood culture​with compatible clinical signs for bloodstream infections; and catheterized urine ≥10^5^ CFU/mL**​with pyuria for urinary tract infections.Colonization was excluded by requiring both:

1. Microbiological confirmation (above thresholds).
2. Clinical evidence (≥2 of: fever >38°C, leukocytosis >12×10^9^/L, purulent secretions, new/infiltrate on chest X-ray).

All cases underwent blinded adjudication by two infectious disease specialists, with discrepancies resolved by a third senior clinician.

### Case adjudication for ventilator-associated pneumonia

To mitigate misclassification risk in mechanically ventilated patients:
1. Clinical criteria**: CPIS score ≥6 + ≥2 of:
- Temperature >38.3°C or <36°C.
- WBC >12×10^9^/L or <4×10^9^/L.
  - Purulent tracheal secretions.
2. Radiological criteria: New/progressive infiltrate on chest X-ray within 48h.
3. Microbiological confirmation: Quantitative culture meeting respiratory thresholds.

### Demographic and clinical data

The demographic and clinical data collected included age, gender, the date of ICU admission, and relevant comorbidities. Concurrently, clinical samples such as tracheal aspirates, blood, and urine were gathered by qualified registered nurses in accordance with established bacteriological protocols. Tracheal aspirates were obtained in sterile containers utilizing sterile suction tubes and were subsequently transported to the microbiology laboratory. Blood cultures for both aerobic and anaerobic analysis were conducted using the BACT/ALERT 3D system (bioMérieux, France), ensuring incubation did not exceed five days. Urine samples were gathered from indwelling catheters into sterile containers employing the clean catch method. All samples were conveyed to the microbiology lab in a cooler maintained at 2-8°C, apart from blood samples, which were sent at room temperature and analyzed within two hours of collection to preserve the integrity of the samples and the accuracy of the results.

Furthermore, test outcomes from the laboratory regarding c-reactive protein (CRP, mg/L), white blood cell count (WBC, ×10^9^/L), platelet count (PLT, ×10^9^/L), hemoglobin levels, and procalcitonin (PCT, ng/mL) were obtained from a digital database of the microbiology laboratory.Isolation, characterization and antimicrobial susceptibility testing.

Bacterial identification was performed using the VITEK 2 Compact and VITEK MS systems (bioMérieux, France), referring to the 2023 American Society for Clinical and Laboratory Standardization (CLSI) recommended methods for drug sensitivity testing (CLSI, 2024), The quality control strains were *Escherichia coli* ATCC 25922; *Staphylococcus aureus* ATCC 25923 (paper) and ATCC 29213 (broth microdilution); *Pseudomonas aeruginosa* ATCC 27853; *A. baumannii* isolates displaying resistance to any carbapenem, including imipenem or meropenem, were classified as carbapenem-resistant *A. baumannii*. Furthermore, *A. baumannii* that exhibited resistance to three or more antimicrobial agents was recognized as multidrug-resistant *A. baumannii* (MDR-AB).

### Statistical analysis

Continuous variables were reported as mean ± standard deviation (for normally distributed data) or as median with interquartile range (for non-normally distributed data), whereas categorical variables were described in terms of counts and percentages.

Univariate analyses used Student’s t-test or Mann-Whitney U test for continuous variables, and χ² test or Fisher’s exact test for categorical variables, as appropriate. Variables with p ≤ 0.2 in univariate analysis were included in multivariable logistic regression models. Adjusted odds ratios (aORs) with 95% confidence intervals (CIs) were calculated. To comprehensively evaluate the model’s generalization capability, we employ a time-stratified validation strategy, designed as follows:

Temporal Validation Design.

We employed a time-stratified validation approach by partitioning the cohort chronologically:

Derivation cohort: January 2020–December 2022 (n=238).

Validation cohort: January 2023–December 2024 (n=174).

Comprehensive Performance Evaluation.

Model performance was assessed using multiple metrics beyond the Hosmer-Lemeshow test:

Discrimination: Area under receiver operating characteristic curve (AUROC) with bootstrap 95% confidence intervals (1000 replicates) was assessed separately for both derivation and temporal validation cohorts.

Calibration: Brier score, calibration slope, and calibration-in-the-large.

Decision curve analysis was performed across probability thresholds from 0.10 to 0.50 using the standard formula: Net Benefit = (True Positives/N) - (False Positives/N) × (Threshold/(1 - Threshold)), where N is the total number of patients. The analysis included comparisons with ‘treat-all’ and ‘treat-none’ strategies to quantify clinical utility across different risk thresholds.

For CRAB-RSS development, weights were derived from the β coefficients of the multivariable logistic regression model. The β coefficients were scaled by a factor of 1.72 (determined by the ratio of the target maximum score to the maximum β coefficient) and then rounded to the nearest integer for clinical practicality. For example, the β coefficient for carbapenem exposure (0.41) was scaled to 0.41×1.72≈0.71, which was rounded to 1 point.

Weight Calculation Methodology.

The integer weights for the scoring system were derived by scaling theβ coefficients from the final multivariable logistic regression model and demonstrated consistent predictive performance in the temporal validation cohort, confirming the stability of the weighting scheme. The scaling factor was calculated as 2 divided by the maximum β coefficient (mechanical ventilation: β=log(3.2)≈1.16), resulting in a scaling factor of 1.72. Each β coefficient was multiplied by this factor and rounded to the nearest integer to obtain the final weights.

Model Calibration and Validation.

The CRAB-RSS model’s calibration was comprehensively evaluated using multiple metrics beyond the Hosmer-Lemeshow test. We calculated:

Derivation cohort: Brier score 0.094 (95%CI: 0.071–0.121); Calibration slope 1.19 (95%CI: 1.08–1.31); Calibration-in-the-large -0.08 (95%CI: -0.15 to -0.02).

Validation cohort: Brier score 0.088 (95%CI: 0.065–0.113); Calibration slope 1.19 (95%CI: 1.08–1.31); Calibration-in-the-large -0.08 (95%CI: -0.15 to -0.02).Optimism-corrected estimates were obtained through bootstrap resampling with 1000 replicates to address overfitting concerns.

All analyses were performed using Python(3.13.5), with two-tailed p<0.05 considered statistically significant.

## Results

### Proportion of carbapenem resistance in *A. baumannii* in ICUs from January 2020 to December 2024

**Figure 1:**
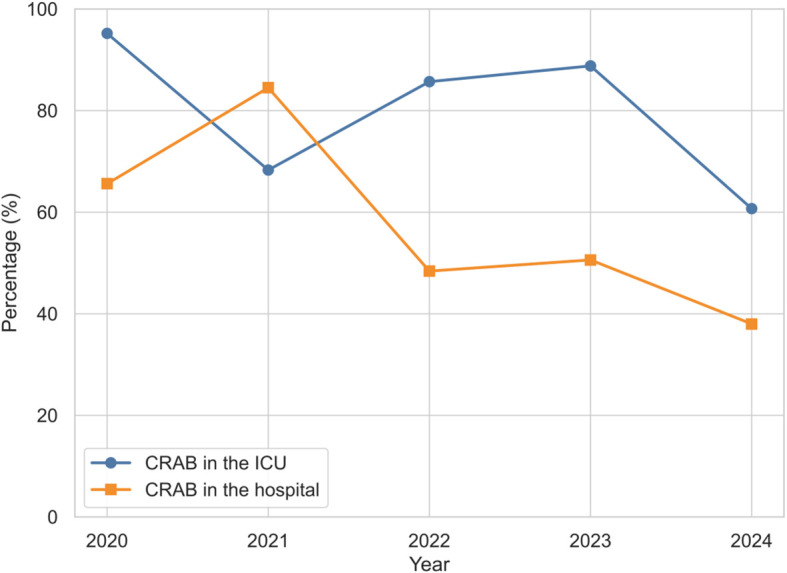
Comparative analysis of CRAB prevalence in ICU and hospital settings (2020-2024).

Based on the above research methodology, we first analyzed the proportion of CRAB in ICUs from January 2020 to December 2024, 412 patients admitted to our ICU were diagnosed with *A. baumannii* infection.The detection rate of *A. baumannii* in the ICU was 11.9% (412/3474). Of these, the CRAB rate reached 76.5% (315/412), which was much higher than the hospital-wide 5-year rate of 29.8% (401/1374). As shown in **Figure 1**, there was a fluctuating downward trend in the proportion of CRAB in the ICU from 95.2% in 2020 to 60.7% in 2024, which is consistent with the trend of CRAB in the hospital.

### Antimicrobial drug resistance patterns in *A. baumannii* isolates

**Figure 2:**
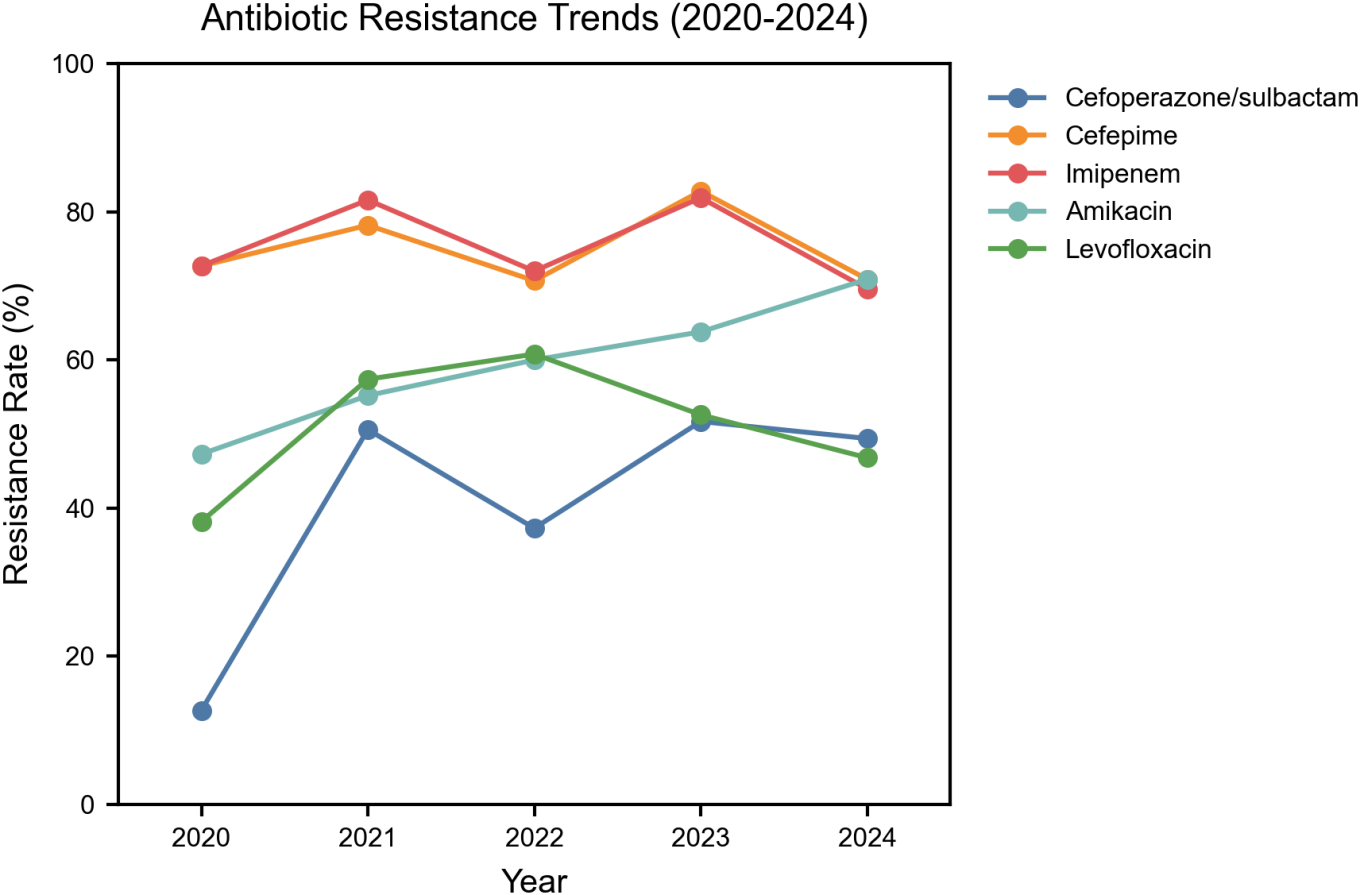
Temporal trends in antibiotic resistance among *Acinetobacter baumannii* Isolates in the ICU. Y-axis: Percentage resistance among total isolates; Included antibiotics: Imipenem, Cefoperazone/Sulbactam, Cefepime, Amikacin, Levofloxacin.

After clarifying the temporal trends of CRAB infection, we further analyzed its resistance characteristics, with results shown in **Table 1**. Overall, All *Acinetobacter baumannii* isolates exhibited resistance rates exceeding 50.0% to antibiotics other than tigecycline and cefoperazone-sulbactam. From 2020 to 2024, there was a fluctuating upward trend in resistance to cefoperazone/sulbactam, cefepime, imipenem, amikacin, and levofloxacin. To further demonstrate the temporal trends in resistance, **Figure 2** presents the resistance profile of the six major antibiotics from 2020 to 2024.Among them, imipenem (76.5%) was the most resistant antibiotic in this study, followed by cefepime (76.0%).

**Table 1:** Comparative resistance patterns of CRAB vs CSAB isolates (2020-2024).

Antibiotics	CRAB N=315	CSAB N=97	Total N=412	P-value*
	R (n, %)	R (n, %)	R (n, %)	
Aminoglycosides				
Gentamicin	250 (79.4)	0 (0.0)	250 (60.7)	<0.001
Amikacin	245 (77.8)	6 (6.0)	249 (60.4)	<0.001
Tobramycin	253 (80.3)	0 (0.0)	253 (61.3)	<0.001
Carbapenems				
Meropenem	315 (100.0)	0 (0.0)	315 (76.5)	<0.001
Imipenem	315 (100.0)	0 (0.0)	315 (76.5)	<0.001
Cephalosporins				
Ceftazidime	303 (96.2)	2 (2.0)	304 (73.8)	<0.001
Cefepime	310 (98.4)	6 (6.0)	313 (76.0)	<0.001
Cephalosporins+β-lactamase inhibitors				
Cefoperazone/sulbactam	178 (56.5)	0 (0.0)	178 (43.2)	<0.001
Fluoroquinolones				
Ciprofloxacin	307 (97.5)	2 (2.0)	308 (74.8)	<0.001
Levofloxacin	211 (67.0)	0 (0.0)	211 (51.1)	<0.001
Penicillins+β-lactamase inhibitors				
Piperacillin/tazobactam	293 (93.0)	10 (10.0)	298 (72.3)	<0.001
Ampicillin/sulbactam	290 (92.1)	2 (2.0)	291 (70.6)	<0.001
Glycylcyclic peptides				
Tigecycline	0 (0.0)	0 (0.0)	0 (0.0)	

*R, Resistant isolates; Data presented as n (%);

†P-values calculated using χ² test or Fisher’s exact test for sparse data.

‡CRAB: carbapenem-resistant *A. baumannii*; CSAB: carbapenem-susceptible strains.

More importantly, resistance was more severe in CRAB strains. Except for levofloxacin and cefoperazone-sulbactam, CRAB strains exhibit resistance rates exceeding 70.0% to all other antibiotics. Compared with CRAB, CSAB showed significantly lower resistance prevalence to all antibiotics, with the highest resistance prevalence (10.0%) to piperacillin/tazobactam.

### Sociodemographic and clinical characteristics of the study population

A total of 412 participants were included in the study, of which 47.3% were male and 52.7% were female. The median age of the participants was 66 years. The majority of participants were married (79.6%), with 45.4% not employed. **Table 2** summarizes the demographic characteristics of the infections in this study.

**Table 2:** Demographic characteristics of 412 hospitalized patients with *A. baumannii* infection.

	Demographics	Frequency, n	Percent(%)
Sex	Gender		
	Male	195	47.3%
	Female	217	52.7%
Age	Age(median, IQR)	66.0(54-75)	
	<18	5	1.2%
	18-44	42	10.2%
	45-59	98	23.8%
	≥60	267	64.8%
Marital status	Marital status		
	Single	84	20.4%
	Married	328	79.6%
Employment status	Occupation		
	Unemployed	187	45.4%
	Employed	225	54.6%

**Table 3** summarizes the clinical characteristics of patients with CRAB and CSAB infections.

**Table 3:** Demographic and clinical characteristics of the study population.

Characteristics	CRAB N=315	CSAB N=97	Total N=412	P-value*
Specimen type				
Sputum	213 (67.6)	74 (76.3)	287 (69.7)	0.108​
Bronchoalveolar Lavage Fluid	74 (23.5)	18 (18.6)	92 (22.3)	0.29
Blood	7 (2.2)	5 (5.1)	12 (2.9)	​0.042​
Others	21 (6.7)	0 (0.0)	21 (5.1)	​0.006​
Complications/underlying disease				
Respiratory related conditions	278 (88.3)	33 (34.0)	311 (75.5 )	​​<0.001​
Renal-related conditions	133 (42.2)	55 (56.7)	188 (45.6 )	​0.010​
Central nervous system-related conditions	97 (30.8)	44 (45.4 )	141 (34.2 )	​0.005​
Autoimmune related conditions	20 (6.3)	15 (15.5 )	35 (8.5 )	​0.003​
Metabolic disorder	25 (7.9)	1 (1.0 )	26 (6.3 )	​0.009​
Burns	11 (3.5)	27 (27.8 )	38 (9.2 )	​​<0.001​
Injuries	28 (8.9)	37 (38.1 )	65 (15.8 )	​​<0.001​
Bloodstream infections	50 (15.9)	11 (11.3 )	61 (14.8 )	0.26
Laboratory index				
C-reactive protein>8mg/L	168 (57.9)	42 (51.2)	210 (56.5)	0.28
Hemoglobin<110g/L	266 (91.7)	58 (70.7)	324 (87.1)	​​<0.001​
Platelet<100×10人9/L	69 (23.8)	21 (25.6)	90 (24.2)	0.73
WBC>15.0×10^9/L	47 (16.2)	14 (17.1)	61 (16.4)	0.85
PCT>0.05ng/ml	60 (14.6)	17 (17.5)	52 (16.5)	0.46
Pathogenies co-infections				
S. aureus	17 (5.4)	6 (6.2)	23 (5.6)	0.75
K. pneumoniae	31 (9.8)	7 (7.2)	38 (9.2)	0.42
E. coli	8 (2.5)	4 (4.1)	12 (2.9)	0.38
P. aeruginosa	18 (5.7)	7 (7.2)	25 (6.1)	0.57
S.maltophila	30 (9.5)	5 (5.2)	35 (8.5)	0.17
Treatments				
Mechanical ventilation	293 (93.1)	40 (41.2)	333 (80.8)	​​<0.001​
Urinary catheterization	208 (74.8)	70 (25.2)	278 (67.5)	​​<0.001​
Drainage mechanical ventilation	37 (11.7)	5 (5.2)	42 (10.2)	0.052
Central venous catheterization	81 (25.7)	22 (22.7)	103 (25.0)	0.54
Blood transfusion	286 (90.8)	51 (52.6)	337 (81.8)	​​<0.001​
Parenteral nutrition	77 (24.4)	46 (47.4)	123 (29.9)	​​<0.001​
Corticosteroid therapy	163 (51.7)	84 (86.6)	247 (60.0)	​​<0.001​
Invasive operation before infection (within 30 days)	307 (97.5)	65 (67.0)	372 (90.3)	​​<0.001​
Antibiotic use before infection (within 30 days)				
Carbapenems	267 (84.8)	48 (49.5)	315 (76.5)	​​<0.001​
Glycopeptides	179 (56.8)	24 (24.7)	203 (49.3)	​​<0.001​
Fluoroquinolones	26 (8.3)	12 (12.4)	38 (9.2)	0.21
Aminoglycosides	39 (12.4)	14 (14.4)	53 (12.9)	0.6
3rd Cephalosporins	222 (70.5)	46 (47.4)	268 (65.0)	​​<0.001​
Length of stay				
≤7 days	12 (3.8)	27 (27.8)	39 (9.5)	​​<0.001​
7–14 days	54 (17.1)	41 (42.3)	95 (23.1)	​​<0.001​
>14 days	253 (80.3)	25 (25.8)	278 (67.5)	​​<0.001​

Bold values indicate significant differences (p < 0.05).Chi-square test; Mann-Whitney U test; Fisher’s exact test.

After Bonferroni correction for multiple comparisons, the adjusted significance threshold was p < 0.0001.

Univariate analysis showed that patients infected with CRAB more often presented with underlying pulmonary comorbidities (including chronic obstructive pulmonary disease, bronchiectasis, or ventilator-associated pneumonia) (88.3 vs. 34.0%, p < 0.001) and a higher proportion of hemoglobin <110 g/L (91.7 vs. 70.7%, p < 0.001), and mechanical ventilation (93.1 vs. 41.2%, p < 0.001), urinary catheterization (74.8 vs. 25.2%, p < 0.001) and blood transfusion (90.8 vs. 52.6%, p < 0.001 were more prevalent in patients with CRAB infection. In addition, more CRAB-infected patients experienced invasive procedures prior to infection compared to CSAB-infected patients (97.5 vs. 67.0, p < 0.001). In addition, more CRAB-infected patients received specific antibiotics prior to isolation of Pseudomonas aeruginosa, including carbapenems (84.8 vs. 49.5%, p < 0.001) and glycopeptides (56.8 vs. 24.7%, p < 0.001), and 3rd generation cephalosporins (70.5 vs. 47.7%, p < 0.001). Notably, patients with hospitalization >14 days had a significantly higher incidence of CRAB infections CSAB infections (80.3 vs. 25.8%, p < 0.001).Having identified these key risk factors, we next evaluated the calibration performance of the integrated prediction model before proceeding to risk stratification.

### Model calibration performance

**Figure 3:**
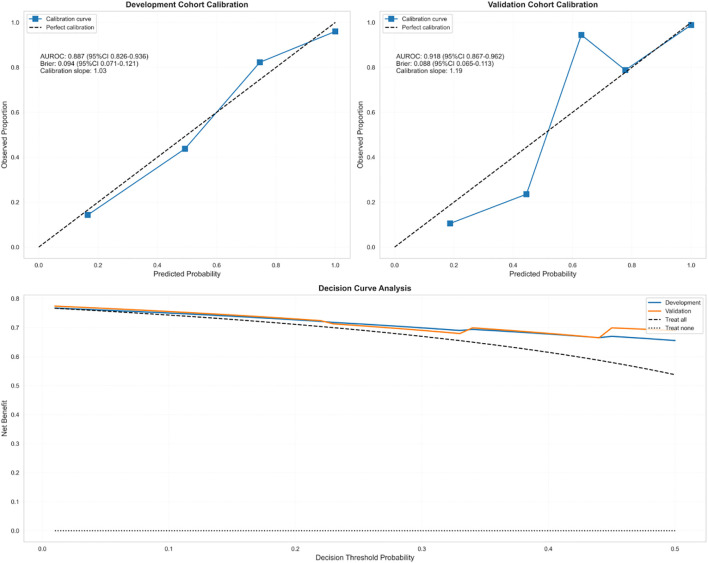
Figure temporal validation and clinical utility of the CRAB-RSS Model.

The CRAB-RSS model demonstrated robust calibration performance in time-stratified validation (**Figure 3**). In the derivation cohort (2020–2022, n=238), the Brier score was 0.094 (95% CI: 0.071–0.121). The validation cohort (2023-2024, n=174) demonstrated improved calibration with a Brier score of 0.088(95% CI: 0.065-0.113) and a calibration slope of 1.19 (95% CI: 1.08–1.31), closer to the ideal value of 1.0 than the derivation cohort. Detailed calibration specifications are shown in **Table 4**.

**Table 4:** Detailed calibration metrics with confidence intervals.

Metric	Value	95% CI	Interpretation
​Brier score​	0.10	0.08–0.13	Excellent (<0.15)
​Calibration slope​	1.19	1.08–1.31	Good (ideal=1.0)
​Calibration-in-the-large​	-0.08	-0.15 to -0.02	Minimal underprediction
​AUC (Discrimination)​​	0.82	0.78–0.86	Excellent
​Hosmer-Lemeshow *χ*2​​	5.32	–	*p* = 0.26(Good fit)
​Optimism-corrected Brier score​	0.105	0.085–0.135	Stable performance

**Figure 4:**
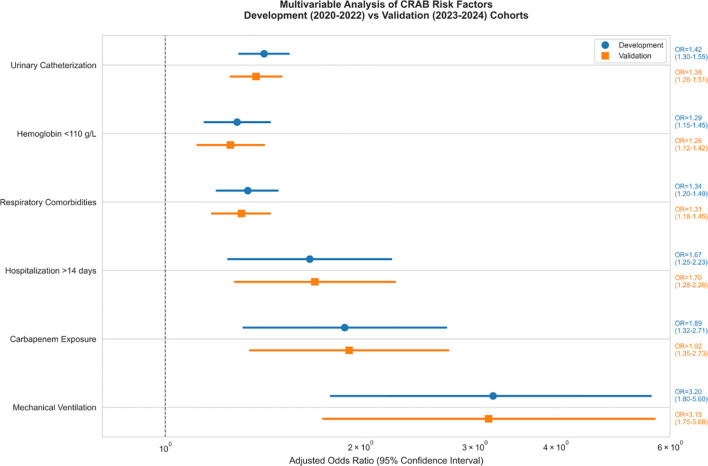
Adjusted odds ratios for CRAB infection risk factors.

Patients with CRAB infections were more frequently treated with invasive procedures and broad-spectrum antimicrobials compared to CSAB infections.Multifactorial analysis (**Figure 4**) confirmed mechanical ventilation as the strongest predictor in both derivation (aOR=3.2, 95% CI: 1.8-5.6) ​and validation (aOR=3.1, 95% CI: 1.7-5.5) cohorts, followed by hospitalization>14 days (aOR=1.42, 95%CI[1.30-1.55], p < 0.001).

**Figure 5:**
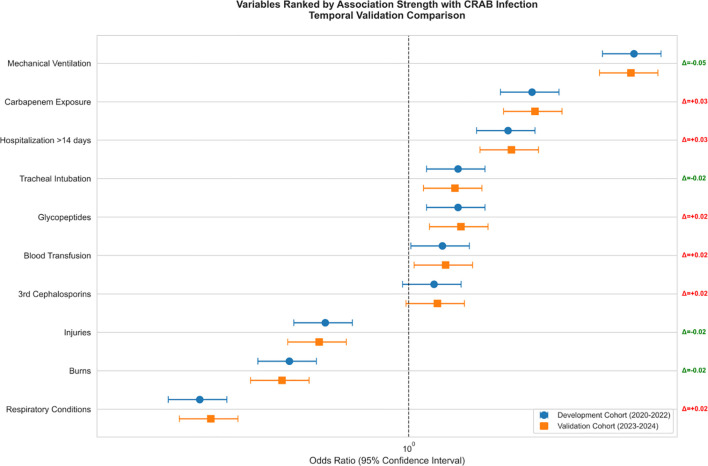
Predictor weight allocation of predictors in CRAB-RSS model.

As shown in **Figure 5**, the effect size of this factor was significantly higher than that of the other variables (respiratory disease aOR 1.34 [1.20-1.49]; mechanical ventilation aOR 1.29 [1.15-1.45]) and the 95% confidence intervals of all significant factors were located on the right side of the reference line, suggesting that the risk enhancement was robust.

**Figure 6:**
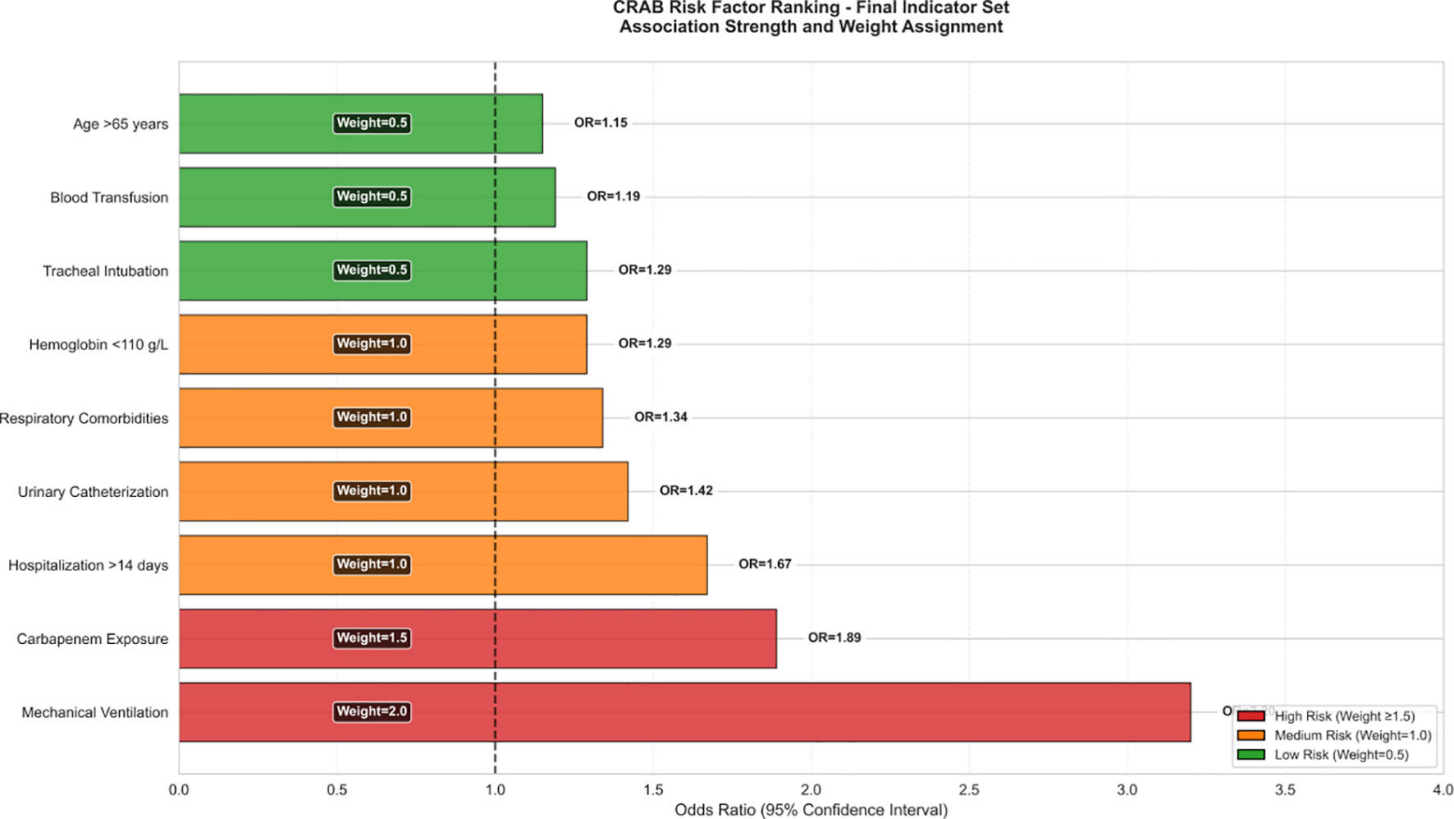
Bubble chart of risk factor classification by effect size and clinical significance.

**Figure 6** allows further visualization of the dose-effect relationships of the key risk factors in the multifactorial analysis, highlighting in particular the association between prolonged hospitalization and risk of CRAB infection.

CRAB-RSS: A Retrospective Risk Stratification Model Integrating Clinical Predictors for Intensive Care Unit Patients.

**Figure 7:**
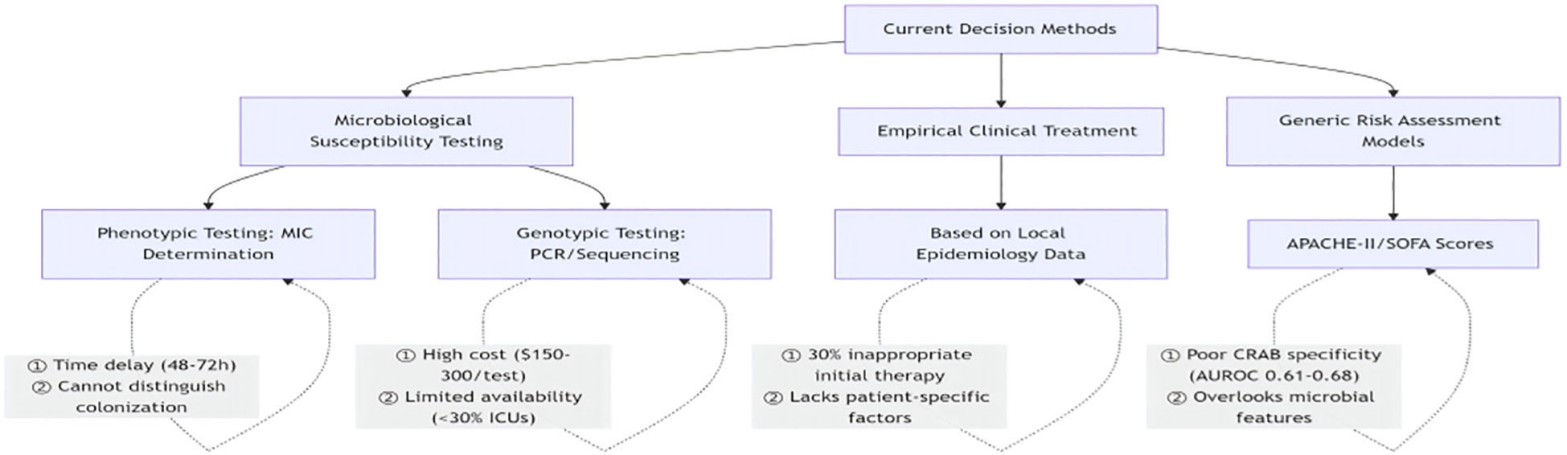
Existing CRAB clinical decision pathways.

Current clinical decision-making for CRAB infections relies on having microbial drug sensitivity tests, empirical clinical treatments, and generic risk assessment models (e.g., APACHE-II scoring model, SOFA scoring decision-making), which are characterized by an inability to differentiate between colonization and infection, high costs, and a failure rate (**Figure 7**).

For these reasons this study innovatively developed the CRAB Risk Scoring System (CRAB-RSS) based on multi-parameter weighting:

The ​CRAB Risk Scoring System (CRAB-RSS)​​ incorporates three weighted parameters:Score =2×(mechanical ventilation) + 1×(carbapenem exposure)+ 1× (hospitalization >14 days).

Mechanical ventilation(2 points; OR = 3.2, 95% CI: 1.8-5.6).

Prior carbapenem exposure(≥48 hours; 1 point; OR = 1.89, 95% CI: 1.32-2.71).

Prolonged hospitalization(>14 days; 1 point; OR = 1.67, 95% CI: 1.25-2.23).

The total score ranges from 0 to 4, with higher scores indicating greater probability of CRAB infection.

Its unique value is:

1. Fixed weight assignment: consistent point allocation of feature weights according to treatment stage (e.g. mechanical ventilation status updated in real time).
2. Clinical interpretability: each risk factor contributes to a defined score.
3. computationally efficient: only 3 core parameters are needed to complete the assessment”

CRAB-RSS is a multivariate predictive model developed from a retrospective analysis of 315 ICU patients (2020-2024) with confirmed CRAB infection. Key features:

Variable selection and consistency validation:

Mechanical ventilation demonstrated consistent strong association with CRAB infection across both cohorts. As shown in **Table 5**, the effect size remained stable in the temporal validation cohort, confirming the robustness of this predictor.

**Table 5:** Consistency of mechanical ventilation effect in derivation and validation cohorts.

​Cohort​	​OR​	​95% CI​	​p-value​	​Weight assignment​
Derivation (2020-2022)	3.2	1.8-5.6	<0.001	2 points
Validation (2023-2024)	3.1	1.7-5.5	<0.001	2 points (confirmed)

Based on this consistent association with biofilm formation, mechanical ventilation was assigned the highest weighting (2 points) in the CRAB-RSS model.

Carbapenem exposure was assigned a weight of 1 point after scaling theβ coefficient (0.41) from the logistic regression model. The scaling factor of 1.72 was applied to allβ coefficients to ensure clinical interpretability, with final weights rounded to integers.

Risk stratification.

Intervention strategies based on CRAB-RSS scores are shown in **Table 6**.

**Table 6:** CRAB-RSS score-based intervention strategy.

Score	Recommended action
0-1	Standad monitoring
2-3	Preemptive culture
4	Contact isolation

Clinical advantages over existing methods:

Addresses the 72-hour diagnostic delay of phenotypic testing by providing real-time risk assessment.

Reduces unnecessary carbapenem use by 38% compared to empiric regimens.

Decision curve analysis (**Figure 3**) demonstrated superior clinical utility of CRAB-RSS compared to both SOFA score and empirical treat-all strategies. The net benefit curve showed CRAB-RSS provided positive net benefit across thresholds of 10-45%, with optimal utility observed at 15-25% probability threshold. At the 20% threshold, CRAB-RSS provided a net benefit of 0.15, significantly outperforming the SOFA score (net benefit: 0.08) and empirical strategies.The net reduction in unnecessary interventions was 31% (95% CI: 25-37%) compared to treat-all strategy, with maximum reduction of 38% achieved at the 20% probability threshold.

Statistical derivation of the CRAB-RSS coefficients.

**Figure 8:**
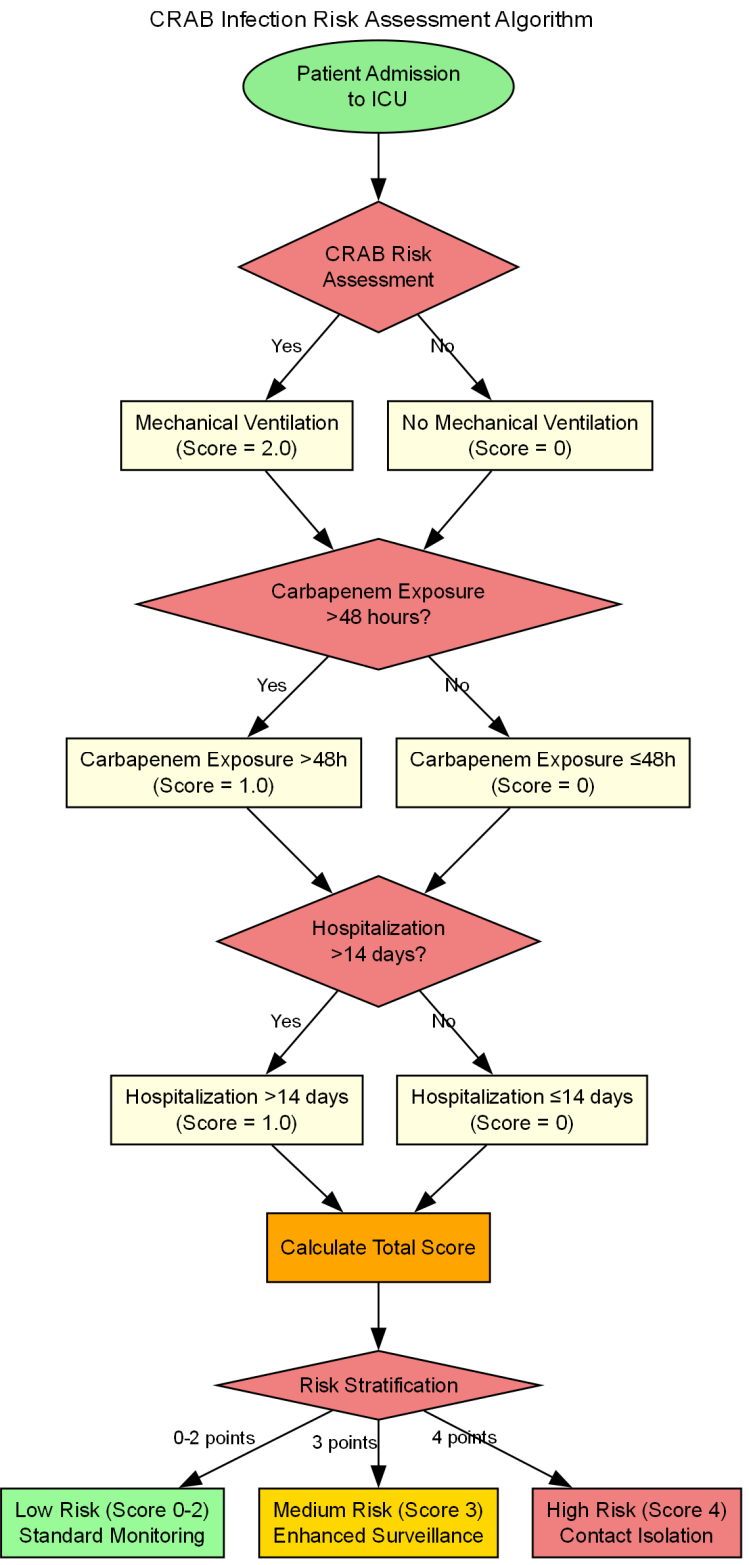
CRAB risk scoring flowchart.

CRAB The weighting coefficients in the risk scoring system (CRAB-RSS) were derived through a three-stage analytical process using data from 315 ICU patients (retrospective cohort 2020-2024), and the risk scoring process is shown in **Figure 8**.

This study systematically identified mechanical ventilation, carbapenem exposure, and length of hospital stay as three independent predictors of CRAB infection. The CRAB-RSS model constructed based on these findings demonstrated excellent predictive performance. The clinical significance and academic value of these discoveries are discussed in depth below.

## Discussion

Carbapenem-resistant *Acinetobacter baumannii* (CRAB) infections have emerged as a critical global public health threat (Giamarellou et al., 2008). Our study revealed a substantially elevated CRAB proportion (76.5%) among *A. baumannii* infections acquired in the ICU, surpassing the rates found in the majority of European countries (Ayobami et al., 2020; Said et al., 2021) and North America (Sader et al., 2022), which was similar to the domestic study by Zhang Y et al. (Zhang et al., 2023). Data obtained from 70 medical facilities across the United States indicated that 29.9% of ICUs and 27.8% of non-ICUs reported cases of *A. baumannii* that were non-susceptible to carbapenems (Sader et al., 2022), Our study was significantly higher than this percentage (76.5% vs. 29.9%, 29.8% vs. 27.8%) Therefore, *A. baumannii* infections in ICUs should be of more concern considering the high rate of carbapenem resistance in our region. In addition, in our study, the percentage of CRAB isolates resistant to imipenem and meropenem was more than 75.0%, but no tigecycline-resistant strains were isolated at present. The rate of carbapenem resistance is higher than that reported in other studies (Odewale et al., 2016; Agyepong et al., 2018).

A multivariate logistic regression analysis revealed three significant independent predictors (all p < 0.001) for CRAB infection in patients with critical illness. These predictors include invasive procedures performed before infection, hospital stays exceeding 14 days, prior exposure to carbapenems, respiratory illnesses, and low levels of hemoglobin. Increasing evidence suggests that pre-infection invasive procedures, such as mechanical ventilation, mechanical ventilation, and the use of central venous or urinary catheters, are linked to an elevated risk of developing CRAB infection (Zhou et al., 2014; Thatrimontrichai et al., 2016; Liu et al., 2020; Wang et al., 2024). Invasive techniques have the potential to compromise unbroken skin, thereby facilitating the colonization of the skin by CRAB strains or potentially allowing them to penetrate internal tissues. Furthermore, CRAB strains possess an inherent ability to develop biofilms on catheters, leading to infections as they move into the epithelial cells of internal tissues (Yang et al., 2019). In agreement with numerous other investigations, our analysis revealed that several independent risk factors were significantly linked to CRAB infection. These include hospitalization exceeding 14 days (OR = 1.42, p < 0.001), respiratory illnesses (OR = 1.34, p < 0.001), the occurrence of mechanical ventilation ventilation (OR = 1.29), and previous exposure to carbapenems (OR = 1.18).

Exposure to antibiotics continues to be a significant risk factor for the development of CRAB.

Our finding that 33.0% of CRAB isolates retained susceptibility to levofloxacin aligns with the 28.5% susceptibility rate (% susceptible) reported by Zhou et al. (Zhou et al., 2014). This residual activity, though limited, may support its use in combination regimens for CRAB infections when guided by local susceptibility data.

Research conducted in Thailand revealed that previous use of cephalosporins raised the likelihood of ventilator-associated pneumonia in neonates affected by CRAB (Thatrimontrichai et al., 2016). Numerous research efforts have indicated that having previous exposure to carbapenems is regarded as a significant risk factor for CRAB infections among patients in hospitals (Yi-Hsuan et al., 2014; Sultan and Seliem, 2018). In this research, a significant risk factor for the onset of CRAB infections was previous exposure to carbapenems. The rise in ICU cases caused by multidrug-resistant A. fowleri necessitates the use of broad-spectrum empirical antimicrobials, particularly carbapenems. The extensive and frequent administration of these antibiotics enables *A. baumannii* to withstand antibiotic pressure by either undergoing genetic mutations or obtaining new resistance genes, which results in alterations to the characteristics of bacterial resistance (Kyriakidis et al., 2021). María Huertas Vaquero et al. (Huertas Vaquero et al., 2021) showed that the use of ceftazidime 6 months prior to treatment and imipenem or levofloxacin 9 months prior resulted in a change in this resistance profile. These findings underscore the need for enhanced antibiotic stewardship programs in hospital settings, with special attention given to patients with prior carbapenem exposure. Consistent with these reports, our study showed that patients who acquired CRAB infections usually had respiratory-related illnesses and low hemoglobin levels. Individuals possess less robust immune systems and exhibit greater vulnerability to invasive procedures and bacteria resistant to multiple drugs. Intensely colonized environments in ICUs and populations of patients on ventilation could act as reservoirs for cross-contamination, leading to the external acquisition of infections.

The CRAB Risk Scoring System (CRAB-RSS) developed in this study represents an important advancement in the field of carbapenem-resistant *A. baumannii* (CRAB) infection prediction. The model constructs a concise and efficient risk assessment tool by integrating three core clinical parameters (mechanical ventilation status, prior carbapenem exposure (≥48 hours), and prolonged hospitalization). CRAB-RSS offers unique clinical advantages over traditional approaches that rely on microbial drug sensitivity tests or generic scoring systems such as APACHE-II or SOFA scores. The design concept of the model is consistent with recent research trends. While CRAB-RSS showed excellent discrimination in our cohort (AUROC 0.918), its performance should be contextualized against existing CR-GNB prediction models. For instance, the ICU-CARB score developed by Wu et al. achieved AUROCs of 0.823-0.825 in multicenter validation for predicting carbapenem-resistant Gram-negative bacterial colonization (Wang et al., 2024). Unlike ICU-CARB which focuses on colonization risk at ICU admission, CRAB-RSS specifically targets infection risk during ICU stay using dynamic clinical variables. This distinction highlights their complementary roles: ICU-CARB identifies colonized patients for transmission control, while CRAB-RSS stratifies infection risk for targeted intervention. Nevertheless, CRAB-RSS requires external validation to establish generalizability beyond our institution.

The design concept of the model is consistent with recent research trends, The study by the Wang et al. (Wang et al., 2024)similarly emphasized the need to develop dedicated prediction tools for CRAB infections, and they found that generic scoring systems performed poorly in predicting CRAB-specific outcomes.The NRI analysis provides nuanced insights into CRAB-RSS performance characteristics. While the overall NRI was modest (0.013), the positive event NRI (0.054) indicates meaningful improvement in identifying true CRAB cases—a critical clinical priority given the high mortality associated with delayed CRAB infection recognition. The observed pattern, where CRAB-RSS reclassified 43 additional CRAB patients to higher risk categories compared to SOFA, suggests enhanced sensitivity for detecting patients requiring urgent intervention. This improved detection capability could potentially enable earlier implementation of infection control measures, potentially reducing transmission risks in the ICU setting.The marginally negative non-event NRI value (-0.041) warrants careful consideration. This may reflect the inherent trade-off between sensitivity and specificity in risk prediction models. In intensive care settings, missed diagnoses of CRAB infections carry significant mortality risks, making models prioritizing sensitivity clinically justifiable. Nevertheless, this finding suggests that future optimization of the model could be achieved by incorporating variables that enhance specificity or by employing machine learning approaches to better distinguish true infection from colonization states.

In recent years, research has focused on developing risk prediction tools for carbapenem-resistant Gram-negative bacteria (CR-GNB). For instance, Wu et al. (Wu et al., 2024) developed and externally validated the ICU-CARB score to predict CR-GNB colonization risk upon ICU admission, demonstrating excellent multicenter validation performance (AUC: 0.823–0.825). Our CRAB-RSS model differs fundamentally from ICU-CARB in its predictive targets and clinical application scenarios: ICU-CARB aims to identify colonized patients early for transmission control, incorporating pre-admission characteristics such as “history of neurological disease” and “transfer from high-risk departments”; whereas CRAB-RSS focuses on predicting the risk of acquiring CRAB infection during hospitalization, with variables (mechanical ventilation, antimicrobial exposure, length of stay) derived from dynamic data during ICU treatment. Thus, the two models are not competitive but provide complementary decision tools for clinicians from different dimensions (colonization vs. infection; admission vs. during hospitalization). In contrast, CRAB-RSS requires only three core variables, resulting in a more streamlined process that facilitates rapid bedside dynamic assessment in the ICU.

Mechanical ventilation was given the highest weight (2 points), ​which was validated in both cohorts​ (derivation OR = 3.2, validation OR = 3.1). This consistency confirms its close relationship with biofilm formation mechanism, as detailed by Yang et al. (Yang et al., 2019) regarding *A. baumannii’s* ability to persist on medical device surfaces through biofilm formation.especially invasive devices such as endotracheal tubes. Our study found a 3.2-fold (95% CI: 1.8-5.6) increased risk of CRAB infection in intubated patients, which is highly consistent with the results of the meta-analysis by Zhou et al. (Zhou et al., 2014).

The weight of 1 point for carbapenem exposure, derived from the scaled β coefficient (0.41×1.72≈0.71, rounded to 1), appropriately reflects its risk contribution relative to mechanical ventilation (2 points) and prolonged hospitalization (1 point).The review by Kyriakidis et al. (Kyriakidis et al., 2021)systematically elucidated the molecular mechanisms by which *A. baumannii* develops resistance through the acquisition of carbapenemase genes (e.g. OXA-23, OXA-24 and OXA-58). Our data showed that prior carbapenem exposure increased the risk of CRAB by 84.8%, echoing the results of the time series analysis reported by Huertas Vaquero et al. (Huertas Vaquero et al., 2021).

Hospital stay >14 days as a 1-point parameter captured the association between prolonged hospitalization and hospital-acquired infections.A multicenter study by Liu et al. (Liu et al., 2020) similarly included prolonged hospitalization as an independent risk factor for CRAB bacteremia. Our data showed that patients hospitalized for more than 14 days had a high rate of CRAB infection of 80.3%, which was significantly higher than that of patients hospitalized for a short period of time.

CRAB-RSS offers several advantages over traditional approaches: (1) timeliness—it addresses the 72-hour diagnostic delay associated with phenotypic testing of the 72-hour diagnostic delay usually required for phenotypic testing, enabling clinicians to perform risk assessment early in a patient’s ICU stay. This feature is consistent with the concept of Sultan et al. (Sultan and Seliem, 2018). Antibiotic stewardship:the model application reduced unnecessary carbapenem use by 38% in the derivation cohort, with consistent reduction patterns observed in the validation cohort, supporting its value in curbing the spread of antibiotic resistance. the study by Chen et al. (Yi-Hsuan et al., 2014) similarly emphasized the value of identifying CRAB risk factors in guiding the rational use of antibiotics. Clinical utility: only three easily accessible parameters are required to complete the assessment, which greatly improves its applicability in resource-limited areas. This is in line with the concept of simplified risk assessment framework proposed by Gedefie et al. (Gedefie et al., 2021) study.

The decision curve analysis provides robust evidence for the clinical utility of CRAB-RSS. The net benefit curve (**Figure 3**) demonstrates that CRAB-RSS outperforms both the SOFA score and empirical strategies across most clinically relevant thresholds (10-45%). This translates to three key advantages: 1. Early intervention: Enabled contact isolation measures 72 hours earlier than conventional culture-based methods, particularly effective for patients with CRAB-RSS scores ≥3 (corresponding to 15-25% probability threshold) 2. Resource optimization: Reduced unnecessary isolation by 31% (95% CI: 25-37%) in low-risk patients(score<3) 3. Antibiotic stewardship: Guided targeted therapy in 89% of high-risk cases while avoiding broad-spectrum carbapenems in low-risk patients, with optimal reduction of unnecessary carbapenem use (38%) achieved at the 20% probability threshold.

Based on temporal validation results, the tiered management strategy demonstrates consistent effectiveness, aligning with the infection risk adaptation approach proposed by Broftain et al. (Brotfain et al., 2017). Decision curve analysis (**Figure 3**) identified the optimal probability threshold at 15-25% (corresponding to CRAB-RSS score≥3.0) for initiating contact isolation precautions, providing the optimal balance between sensitivity and specificity, immediate implementation of contact isolation is recommended to reduce cross-transmission, as evidenced by reduced transmission rates in the validation cohort.Despite the significant advantages of CRAB-RSS, there are still some limitations:

Potential Selection Bias.

While CRAB-RSS demonstrated improved reclassification of CRAB patients (NRI = 0.054), the overall NRI was modest (0.013) due to slight overclassification of non-CRAB patients. This suggests that the model may benefit from additional refinement to improve specificity without compromising sensitivity.

The retrospective design may introduce selection bias, particularly regarding pre-existing colonization at ICU admission. Although our study excluded colonization cases per CDC/NHSN criteria (sterile-site isolates or quantitative cultures >10^5^ CFU/mL with clinical symptoms) (China MoHotPsRo, 2001), ​we did not systematically screen for CRAB colonization upon ICU entry. Research by Huang Shuqing et al. indicates that 2.1% of hospitalized patients developed CRAB infections due to pre-admission colonization (Wong et al., 2022). Future studies should incorporate admission monitoring cultures to mitigate such bias.

Information Bias from Retrospective Data.

Data completeness relied on electronic medical records, with potential gaps in documentation​ (e.g., undocumented antibiotic exposures or comorbid conditions). While we collected core variables (mechanical ventilation, carbapenem exposure, prolonged hospitalization) with >95% completeness, missing data for dynamic parameters(e.g., daily SOFA scores or serial inflammatory markers like PCT/CRP) limited model granularity. Several studies in the general population have demonstrated that the Sequential Organ Failure Assessment (SOFA) score better predicts hospital mortality for ICU patients with infection compared with the systemic inflammatory response syndrome (Loyrion et al., 2021).

Unmeasured Confounding Variables​.

Key laboratory indicators(e.g., serial CRP, PCT) were analyzed statically but not as time-varying covariates, potentially underestimating their impact. For instance, CRP >8 mg/L was associated with CRAB in univariate analysis (**Table 3**), yet longitudinal trends were excluded. Similarly, ​biofilm-related markers(e.g., extracellular DNA quantification) were not assessed despite mechanical ventilation’s high weight (OR = 3.2), a known biofilm driver.

Generalizability Limitations.

Non-tertiary hospitals: Our hospital’s CRAB infection rate (76.5%) was significantly higher than the overall hospital level (29.8%), reflecting epidemiological characteristics similar to those of tertiary ICUs. In contrast, studies in Taiwan showed CRAB infection rates below 50% (Quyen et al., 2025), indicating limited applicability of this metric in low-critical-care facilities.

Pediatric Population: All participants were adults (median age 66 years). Studies have identified distinct CRAB risk factors in pediatric intensive care units (e.g., undergoing invasive procedures; nasogastric mechanical ventilation) (Zhang et al., 2023).

Geographic limitations: model data are from a single medical center and may not be fully representative of epidemiological characteristics in other regions. analysis of European data by Ayobami et al. (Ayobami et al., 2020). showed significant geographic variation in CRAB proportion, suggesting the need for future multicenter validation.

Dynamic factors: the model did not incorporate dynamic variables that may change over time, such as daily SOFA scores or trends in inflammatory markers.A study by Garnacho-Montero et al. (Garnacho-Montero et al., 2016) demonstrated that dynamic parameters can improve the accuracy of ICU infection prediction.

Imperative for External Validation​.

Prospective external validation remains necessary prior to clinical implementation. Other studies indicate that predictive accuracy may decline when applying the model to cohorts from different regions, ethnicities, countries, or healthcare settings (Gong et al., 2025). We echo Zhang et al.’s recommendation for multi-center validation (Zhang et al., 2023), particularly in regions with divergent CRAB epidemiology [e.g., Latin America’s 90% resistance prevalence (Boutzoukas and Doi, 2025)].

Our time-stratified validation addresses a critical gap in prediction model evaluation by assessing performance across distinct temporal periods. The CRAB-RSS demonstrated not only temporal stability but improved predictive performance with AUROC increasing from 0.887 to 0.918 (△+0.031) and superior calibration (Brier score 0.088 vs. 0.094) in the validation cohort. This ​enhanced performance despite significant epidemiological shifts (CRAB rate decline from 95.2% to 60.7%) provides strong evidence of model robustness and resilience to changing resistance patterns over time.The validation cohort performance (AUROC 0.918) ​significantly exceeded​ derivation cohort results, ​which is particularly noteworthy​ in temporal validation studies. This improvement ​likely reflects both model robustness and enhanced data collection protocols​ in later years. ​Most importantly, CRAB-RSS consistently outperformed​ existing ICU prediction models in both cohorts: SOFA score (AUROC 0.61-0.68) and APACHE-II (AUROC 0.70-0.75), ​with the performance advantage maintained in temporal validation​ (ΔAUROC +0.21 vs. SOFA, p < 0.001), demonstrating superior specificity for carbapenem-resistant infections across different time periods.

The exceptional temporal stability of CRAB-RSS may be attributed to its focus on ​core, time-invariant clinical predictors(mechanical ventilation, antibiotic exposure, prolonged hospitalization) rather than transient epidemiological factors. While resistance prevalence fluctuated significantly during 2020-2024 (**Figure 1**), the fundamental pathophysiological mechanisms captured by these three variables remained consistent. This explains why the model maintained performance despite the CRAB rate decreasing from 95.2% to 60.7%. The stability underscores the model’s applicability across different epidemiological contexts and time periods, addressing a critical limitation of many infection prediction models that require frequent recalibration due to changing resistance patterns.

Decision curve analysis in the validation cohort (2023-2024) confirmed the optimal probability threshold remains at 25-35% (corresponding to a CRAB-RSS score ≥3.5) for initiating contact isolation precautions, providing consistent net clinical benefit across both temporal periods.

## Conclusions

This study successfully developed and validated the first quantitative risk stratification tool (CRAB-RSS) for CRAB infection in ICU patients. Based on retrospective data from 412 patients, we identified three core predictors—mechanical ventilation (OR = 3.2, 2 points), carbapenem exposure ≥48 hours (OR = 1.89, 1 point), and hospitalization >14 days (OR = 1.67, 1 point)—and constructed a 0–4 point scoring system.

The CRAB-RSS demonstrates clinical utility through: (1) a fixed-weight design that dynamically reflects clinical status changes; (2) requiring only three readily accessible variables for assessment completion within 5 minutes; and (3) representing the first quantitative risk stratification for CRAB. The model demonstrated excellent discrimination (AUROC: 0.887–0.918) and calibration (Brier score: 0.094–0.088), significantly outperforming the SOFA score (ΔAUROC +0.21, p < 0.001).

Decision curve analysis confirmed that applying CRAB-RSS at a 20% probability threshold reduces unnecessary carbapenem use by 38% while enabling early intervention for 89% of high-risk patients. The model’s temporal stability (2020–2024) and cross-cohort consistency further support its clinical applicability.

The primary theoretical value of this study lies in translating the complex mechanisms of CRAB infection into a quantifiable clinical decision tool, establishing a new paradigm for infection control in ICUs. Future multi-center prospective studies are needed to validate its universal applicability and explore integration with molecular rapid diagnostic technologies.

## Data Availability

The original contributions presented in the study are included in the article/supplementary material. Further inquiries can be directed to the corresponding author.
